# The Longitudinal Aging Study Amsterdam: cohort update 2019 and additional data collections

**DOI:** 10.1007/s10654-019-00541-2

**Published:** 2019-07-25

**Authors:** Emiel O. Hoogendijk, Dorly J. H. Deeg, Sascha de Breij, Silvia S. Klokgieters, Almar A. L. Kok, Najada Stringa, Erik J. Timmermans, Natasja M. van Schoor, Elisabeth M. van Zutphen, Marleen van der Horst, Jan Poppelaars, Priyanta Malhoe, Martijn Huisman

**Affiliations:** 1 Department of Epidemiology and Biostatistics, Amsterdam Public Health Research Institute, Amsterdam UMC - Location VU University Medical Center, Amsterdam, The Netherlands; 2 Department of Psychiatry, Amsterdam Public Health Research Institute, Amsterdam UMC - Location VU University Medical Center, Amsterdam, The Netherlands; 3 GGZ InGeest Specialized Mental Health Care, Amsterdam, The Netherlands; 4 Department of Sociology, Faculty of Social Sciences, Vrije Universiteit, Amsterdam, The Netherlands

**Keywords:** Longitudinal studies, Cohort studies, Netherlands, Epidemiology, Aging, Genotyping data, Older migrants, Study design

## Abstract

The Longitudinal Aging Study Amsterdam (LASA) is a prospective cohort study of older adults in the Netherlands, initially based on a nationally representative sample of people aged 55–84 years. The study has been ongoing since 1992, and focuses on the determinants, trajectories and consequences of physical, cognitive, emotional and social functioning. Strengths of the LASA study include its multidisciplinary character, the availability of over 25 years of follow-up, and the cohort-sequential design that allows investigations of longitudinal changes, cohort differences and time trends in functioning. The findings from LASA have been reported in over 600 publications so far (see www.lasa-vu.nl). This article provides an update of the design of the LASA study and its methods, on the basis of recent developments. We describe additional data collections, such as additional nine-monthly measurements in-between the regular three-yearly waves that have been conducted among the oldest old during 2016–2019, and the inclusion of a cohort of older Turkish and Moroccan migrants.

## Introduction

The Longitudinal Aging Study Amsterdam (LASA) is a prospective cohort study among older adults in the Netherlands. The study started in 1992, initiated by the Dutch Ministry of Welfare, Health and Culture (currently Ministry of Health, Welfare and Sports), and is still ongoing. The primary objective of LASA was to study the determinants, trajectories and consequences of (changes in) functioning in four domains: physical, cognitive, emotional and social functioning [1, 2]. LASA is one of the few longitudinal studies of older adults in the Netherlands [3, 4], and worldwide among the few studies covering a broad range of functional domains. The main strengths of the study include its multidisciplinary character, the availability of over 25 years of follow-up, and the inclusion of refresher cohorts of young older adults at 10 and 20 years after the start of the study. This structure provides unique opportunities to investigate longitudinal trajectories, cohort differences and time trends in functioning of older adults [5, 6].

In this paper, we briefly describe the design of LASA and provide an update of the methods. In particular, this paper describes the additional data collections that have not been described in detail previously, such as data collected in telephone interviews, additional nine-monthly measurements among the oldest old, genetic data, environmental data, and the inclusion of a migrant cohort.

## The design of LASA

The LASA cohort was initially based on a representative sample of older adults aged 55–84 years (born between 1908 and 1937) from three regions in the Netherlands. These three regions (areas in and around the cities of Zwolle, Oss and Amsterdam) were selected to obtain an optimal representation of the Dutch older population. The regions cover the predominantly protestant northeast, the largely catholic south and the more secularized western part of the Netherlands, and include both urbanized and rural areas. The initial LASA sample consists of people who first participated in the NESTOR study on Living Arrangements and Social Networks of older adults (LSN) [7]. The sample for the LSN study was randomly selected from municipality registers in 1992, with an oversampling of the oldest old and men. This oversampling was done to ensure that there would be reasonable numbers of oldest old and very old men, even after several years of follow-up. The initial sample of the LSN study consisted of 3805 persons, which corresponds to a response rate (defined as the number of complete and partial interviews, divided by the total number of eligible persons in the sample plus a fraction of those persons who were in the sample but of whom eligibility could not be determined) of 60%. The cooperation rate (defined as the number of completed interviews divided by the total number of contacted eligible persons) was 62%.

**Fig. 1:**
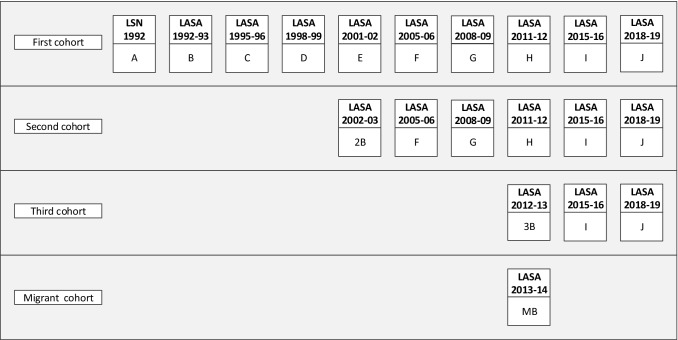
Overview of LASA cohorts and measurement waves

**Fig. 2:**
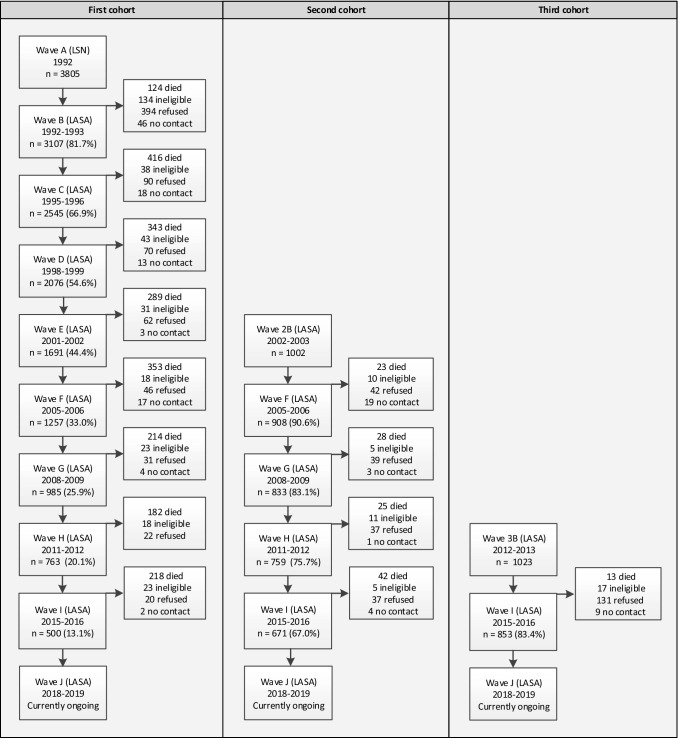
LASA study flowchart

On average, 11 months after the LSN interview (wave A), respondents were invited to participate in the first wave of LASA (wave B, n = 3107). The response rate was 85% and the cooperation rate was 89%. Since the 1992 LSN measurement wave, there have been nine LASA measurement waves to date (Figs. 1, 2). At the eighth measurement wave (wave I), approximately 23 years after the start of the study, a total of 500 respondents from the original cohort were still participating.

Two refresher cohorts were added to the original sample in 2002–2003 and 2012–2013, exactly 10 and 20 years after the start of the LASA study. Figure 2 and Table 1 show the sample sizes for the three cohorts across all measurement waves and by interview type. The second cohort (included in 2002–2003) consisted of 1002 men and women born between 1938 and 1947 (cooperation rate = 62%), and the third cohort (included in 2012–2013) consisted of 1023 men and women born between 1948 and 1957 (cooperation rate = 63%). In follow-up measurements after the baseline measurement, respondents from these new cohorts were merged with those from the original cohort. At the end of 2019, the data collection of the most recent measurement wave (wave J), consisting of the remaining respondents from all three cohorts, will be completed.

**Table 1:** LASA interview type by measurement wave

Wave	Year	Cohort^a^	Age range	n	n by interview type		
					Face-to-face	Telephone	
					Main interview (medical interview)	Respondent	Proxy
B	1992–1993	Cohort 1	55–84	3107	3107 (2671)	–	–
C	1995–1996	Cohort 1	57–88	2545	2302 (1509)^b^	164	79
D	1998–1999	Cohort 1	60–91	2076	1874 (1382)^c^	127	75
E	2001–2002	Cohort 1	63–94	1691	1474 (1307)	122	95
2B	2002–2003	Cohort 2	55–64	1002	1002 (919)	–	–
F	2005–2006	Cohort 1, 2	57–98	2165	1908 (1805)	140	117
G	2008–2009	Cohort 1, 2	60–100	1818	1601 (1494)	117	100
H	2011–2012	Cohort 1, 2	63–104	1522	1308 (1212)	99	115
3B	2012–2013	Cohort 3	55–64	1023	1023 (889)	–	–
MB	2013–2014	Migrant cohort	55–64	478	478 (344)	–	–
I	2015–2016	Cohort 1, 2, 3	57–102	2024	1770 (1642)	148	106

^a^Birth years Cohort 1: 1908–1937, Cohort 2: 1938–1947, Cohort 3: 1948–1957, Migrant cohort: 1948–1957

^b^In 1995–1996, only people born before 1931 were asked to participate in the medical interview

^c^In 1998–1999, only people born before 1931 plus a control group of remaining birth years were asked to participate in the medical interview

The LASA study is conducted in line with the Declaration of Helsinki and received approval by the medical ethics committee of the VU University Medical Center (IRB numbers: 92/138, 2002/141, 2012/361, and 2016.301).

## Data collection

### Face-to-face interviews

Respondents are visited every 3 to 4 years at home by trained interviewers who collect the data. All interviews (if respondents agree) are audio-recorded for quality checks. Measurements are performed for each of the four domains of functioning: physical, cognitive, emotional and social functioning. The data collection includes both questionnaires and clinical tests. An overview of the main predictors and outcome measures in LASA has been published before [2]. Detailed descriptions of the measurements can be found on the LASA website (www.lasa-vu.nl). The data collection consists of three elements: a main interview, a self-administered questionnaire and a medical interview. The main interview takes, on average, almost 2 h to complete. An abbreviated interview may be offered to the respondent when a full interview is too burdensome to complete. At the end of the main interview, respondents are asked to fill out a self-administered questionnaire, which is left at the respondent’s home in print, or can be accessed online (as of 2015). Respondents are asked to hand in this questionnaire during the medical interview, to return it by postal mail, or to complete the questionnaire online. Respondents are also invited to participate in a subsequent medical interview. After consent, a separate visit is scheduled, in which additional questions are asked and clinical measurements are performed. This medical interview takes on average 1 h and a half to complete. Respondents who score highly on the symptom checklists of depression or anxiety are invited for an additional diagnostic psychiatric interview. At specific measurement waves (waves B, C, 2B, G and 3B) blood samples were obtained from respondents who participated in the medical interview [2].

### Telephone data

Since LASA measurement wave C (1995–1996), a telephone interview is offered to those respondents who refused to participate in a full or an abbreviated face-to-face interview. The telephone interview takes approximately 15 min and includes a selection of key indicators of functioning (see Table 2). While this interview mode was initially intended to obtain information from proxy respondents, it turned out that quite a few respondents were willing to answer the telephone questions themselves even though they could not participate in the face-to-face interview. Across all measurement waves up to 2015–2016 (wave I), the majority of telephone interviews was in fact conducted with respondents: 4.4% of all interviews were done by telephone with respondents, versus 3.3% by telephone with proxies.

**Table 2:** Measures in telephone interview

Measure	Availability	Measurement waves							
Demographic data (housing, partner status)	Respondent + proxy	C	D	E	F	G	H	I	J
Chronic diseases	Respondent + proxy	C	D	E	F	G	H	I	J
Functional limitations	Respondent + proxy	C	D	E	F	G	H	I	J
Senses (vision, hearing)	Respondent + proxy	C	D	E	F	G	H	I	J
Fractures/falls^a^	Respondent + proxy	C	D	E	F	G	H	I	J
Weight change	Respondent							I	J
Depressive symptoms (CES-D)	Respondent	C	D	E	F	G	H	I	J
	Proxy	C	D	E					
Cognitive decline (IQCODE)	Proxy	C	D	E	F	G	H	I	J
General cognitive functioning (MMSE, short version)	Respondent		D	E	F	G	H	I	J
Salience of religion	Respondent + proxy	C							
End of life (advance directives)	Respondent		D						
Use of care	Respondent + proxy	C	D	E	F	G	H	I	J
Needs assessment (facilities, social services, care)	Respondent + proxy					G	H	I	J
Loneliness (De Jong Gierveld loneliness scale, short version)	Respondent						H	I	J
Employment and retirement	Respondent							I	J

*CES-D* Center for Epidemiologic Studies Depression scale, *IQCODE* Informant Questionnaire of Cognitive Decline in The Elderly, *MMSE* Mini Mental State Examination

^a^Information on falls is only available at wave C, D and J

Across all measurement waves, respondents with telephone data were older than respondents with face-to-face data, less often lived with a partner and more often lived in a care institution. Net of age, there were no differences in level of education. Respondents with telephone data were also less healthy in terms of number of chronic diseases, functional limitations and self-rated health than those with face-to-face data. Besides being too frail, one reason for respondents to refuse a face-to-face interview, is that they may be too busy. In 2015–2016 (wave I), respondents aged 74 years or younger were asked about paid work and number of hours of work. It turned out that telephone respondents indeed had paid work relatively often (38.6% vs. 32.1% among face-to-face respondents) and that those who worked also worked relatively long hours (32.8 h vs. 29.3 h among face-to-face respondents). Both differences were, however, not statistically significant. In order to better reflect the variation in the population, it is recommended that telephone data are used in addition to face-to-face data, whenever the research question allows it. This helps to minimize selection bias.

## Attrition and representativeness

Attrition of respondents over time is a key concern for longitudinal studies, which may affect the representativeness of the sample [1]. The main reason for drop-out in LASA is mortality [1, 2, 8]. If mortality in the LASA study differs from mortality in the general population, this could be a threat for the representativeness of the study, as it could lead to an increasingly selective sample. Therefore, analyses were performed to compare mortality among LASA respondents with mortality in the Dutch general population. Data on mortality in the general population by sex, year and age group were obtained from Statistics Netherlands [9]. The results show that mortality was slightly higher in the general population than in the LASA sample, but for most groups differences did not exceed 1 percent point (Table 3). Thus, it can be concluded that mortality in the LASA study is not substantially different from mortality rates in the Dutch general older population.

**Table 3:** Mortality among LASA respondents compared to the Dutch general population

	Weighted sum of difference (LASA minus Dutch population)^a,b^	
	Men	Women
Total	− 0.73	− 0.51
By year		
1994	− 0.53	− 0.54
1997	− 0.75	− 0.50
2000	− 1.00	− 0.81
2004	− 0.72	− 0.57
2007	− 0.38	− 0.09
2010	− 0.94	− 0.45
2014	− 0.89	− 0.63
By age		
60–64 years	− 0.37	− 0.16
65–69 years	− 0.40	− 0.06
70–74 years	− 0.37	− 0.45
75–79 years	− 1.03	− 0.97
80–85 years	− 1.74	− 1.09

^a^Expressed in percent point difference. All differences were summed, and weights were applied for the number of LASA-respondents in each group

^b^We estimated 1-year mortality in LASA by dividing the percentage that died between subsequent measurement waves by three, since the interviews were held with 3-year intervals. Exceptions were the interval between wave E/2B (2001–2003) and wave F (2005–2006), which was on average 3.7 years, and the interval between wave H/3B (2011–2013) and wave I (2015–2016), which was on average 3.6 years. For each year (t), the percentage that died in each age group (X) between two successive waves in LASA was calculated as follows: number of deaths in age group X (at the time of death) between the two waves/number of deaths + number of survivors in age group X (at January 1 in year t, where t is the mid-year between two waves)

A further indication of the representativeness of the LASA cohorts is obtained by comparing the frequency of work participation between LASA and the general population as assessed by Statistics Netherlands, for the age group 55–64 years. We performed this comparison for the baseline wave of the second (wave 2B, 2002–2003) and third cohort (wave 3B, 2012–2013), for men and women and for three levels of education. Work participation was defined as having paid work for 1 h or more. The difference between frequencies in LASA and the general population was 2.6 percent points on average, thus showing good correspondence. Across subgroups, the differences ranged from 0.2 to 6.9 percent points. The larger deviations were observed for women and the lower educated, which may be attributed to the greater precariousness of work in these groups, so that their work participation is more likely to fluctuate over time.

## Methods update

### Additional nine-monthly measurements among the oldest old

The oldest old are in a stage of life in which changes in functioning can occur more rapidly and more catastrophically than earlier in life. For example, cognitive decline markedly accelerates during the last years of life [10]. Therefore, it is important to accurately monitor trajectories of functioning and changes in functioning in this age group. At the same time, there have been recent changes in policy in the Netherlands that may particularly affect the oldest old. As of 2015, the Social Support Act (WMO) directs municipalities to provide support for people with functional limitations, including instrumental support at home, home care and social care, which was previously regulated by the national government. This may lead to variations in care provision between different municipalities. In addition, the Long-term Care Act for residential care (WLZ), and the Care Insurance Act for personal and nursing home care at home (ZVW) were implemented. In these acts, thresholds for access to care were raised, making it more difficult to be eligible for residential care, which can lead to an increased reliance on informal care and privately paid care. The absolute increase in the number of oldest old in the community, the rapid changes in functioning among the oldest old and the recent policy changes were the main reasons for conducting an ancillary study among the oldest LASA respondents with increased density of measurements.

Three additional nine-monthly measurements were performed between the regular LASA measurements in 2015–2016 (wave I) and 2018–2019 (wave J). Thus, together with these regular measurement waves, data from five consecutive nine-monthly measurements will become available for studying changes and trajectories of the four domains of functioning. All persons aged 75 years and over (born before 1941) were invited to participate in this ancillary study (n = 686). In total, 601 persons agreed to participate (87.6%). At the first additional measurement (wave I—v1), 442 (73.5%) participated in a face-to-face home interview and 159 (26.5%) participated in a telephone interview (61 with respondent and 98 with proxy). The topics included in the interview, as well as the response rates for each additional nine-monthly measurement, are presented in Table 4. Respondents who had a face-to-face interview were asked to fill out a one-week calendar to study changes in pain, use of pain medication, mood, sleep, social contacts and appetite on a daily basis. Respondents were asked to return the calendar by postal mail.

**Table 4:** Ancillary study: additional nine-monthly measurements among the oldest old (born before 1941)

Response	Wave I—v1	Wave I—v2	Wave I—v3
Date range interviews	July 2016–July 2017	April 2017–April 2018	January 2018–January 2019
Invited, n	686	601	550
Participated, n (%)	601 (87.6)	550 (91.5)	507 (92.2)
Age, mean (SD)	83.0 (5.4)	83.4 (5.2)	83.8 (4.9)
Data available			
Face-to-face interview, n	442	410	364
Calendar data, n^a^	387	368	325
Telephone interview Respondent, n	61	55	59
Telephone interview Proxy, n	98	85	84
*Measures*			
Face-to-face interview	Demographic data, gait speed, grip strength, chronic diseases, self-rated health, functional limitations, homecare/informal care, care needs, healthcare use, depressive symptoms (CES-D, short version), falls and fractures, memory complaints, cognitive functioning (MMSE, coding task), loneliness (De Jong Gierveld loneliness scale, short version), weight measurement, self-reported weight change, physical activity, pain, end of life care and preferences, and partner health		
Calendar data	One-week calendar, with questions on pain (1–10; severe pain-no pain), use of pain medication (yes/no), mood (1–10; very bad-very good), sleep (1–10; very bad-very good), social contact (number of people), and appetite (1–5; very bad-very good) on a daily basis		
Telephone interview	Demographic data, chronic diseases, self-rated health, functional limitations, homecare/informal care, care needs, healthcare use, depressive symptoms (CES-D, short version), falls and fractures, memory complaints, cognitive functioning (MMSE, short version), loneliness (De Jong Gierveld loneliness scale, short version), self-reported weight change, physical activity, pain, end of life care and preferences, and partner health		

^a^Calendar data is only available for those participating in the face-to-face interview

### Genetic data

Blood samples from respondents participating in the LASA medical interview in 1995–1996 (wave C), 2002–2003 (wave 2B), 2008–2009 (wave G) and 2012–2013 (wave 3B) were used to obtain genetic data. In the first and second cohort, DNA was isolated from buffy coats in wave C and wave 2B and from full blood samples in wave G. For respondents from whom both full blood samples and buffy coats were available, the full blood samples were used to extract DNA. In the third cohort, full blood samples drawn at baseline (wave 3B) were used for DNA isolation. In all samples, DNA was extracted using standard procedures.

In 2016–2017 genotyping array data were generated for respondents who had blood samples available. First, a batch of around 600 samples was genotyped using Axiom-NL array [11] (Affymetrix Inc., Santa Clara, CA, USA) at the Avera Institute for Human Genetics, Sioux Falls, SD, USA. Then, another 1880 samples from the first, second and third cohort were genotyped using Infinium Global Screening Array-24-v.1.0 (GSA) (Illumina Inc., San Diego, CA, USA) at the Genetic Laboratory, Department of Internal Medicine, Erasmus MC, Rotterdam, the Netherlands. Due to technical differences, quality control and imputation were performed separately for each array. For both arrays, quality control was performed using Ricopili (RICOPILI: Rapid Imputation for COnsortias PIpeLIne) [12], an established tool developed by the Psychiatric Genomics Consortium [13, 14]. Samples with sex mismatch, duplicate samples, excess heterozygosity and call rate < 0.98 were removed after quality control. Single nucleotide polymorphism (SNPs) with call rate < 0.98 and minor allele frequency < 0.01 were also excluded. Ancestry related principal components for each array were calculated. Samples of non-European ancestry were identified and later removed using the 1000 Genome data as reference. Then, 10 principal components were re-calculated for each array for European ancestry respondents. The data was further checked for relatedness between respondents.

Overall, genotyping array data are available for European-ancestry, non-related respondents (n = 2279); from cohort 1 (n = 1081, of which n = 590 genotyped with Axiom-NL and n = 491 genotyped with GSA), from cohort 2 (n = 631) and from cohort 3 (n = 567). After quality control, both arrays were separately used to impute the data using as reference the Haplotype Reference Consortium panel version 1.1 [15]. Imputation for autosomal chromosomes (chr1-22) was done using Minimac 3 and was facilitated by the Michigan Imputation Server [16].

Genotyping data available in LASA can be used in candidate gene studies, to build polygenic risk scores for complex traits and in genome-wide association studies (GWAS). In combination with the rich pool of physical, cognitive, emotional and social phenotypes in LASA, genotyping array data are a valuable resource for gene-environment interaction studies. LASA has been included in GWAS meta-analyses from the CHARGE and GEFOS consortia recently as well as other collaborations [17–19].

Exome chip data and a number of SNPs in candidate genes are available for a subsample of respondents in the first cohort (n = 1193) (see for example [20]). Furthermore, genotyping of Apolipoprotein E gene is also available for a subset of respondents in the second cohort (n = 753). An overview of the exome chip data and candidate SNPs has been previously published [2].

### Chronic disease and composite algorithms

In LASA, various sources of data on chronic disease are available, including self-reported chronic diseases, medication use in the two weeks before the interview and diagnoses from general practitioner (GP) records. Since none of these data sources are the gold standard to measure disease status, chronic disease ascertainment algorithms were developed. In these algorithms, data from different sources are combined [21]. Algorithms were constructed for seven cardiovascular diseases (CVD) (angina pectoris, myocardial infarction, coronary artery disease, congestive heart failure, peripheral arterial disease, cardiac arrhythmia and cerebrovascular accident), diabetes mellitus and osteoarthritis [22–24]. Using these algorithms, respondents were categorized as definitely, possibly or not having a disease, contradictory data, or as missing when there are no data available. An illustrative example of diabetes mellitus disease status according to the chronic disease algorithm and other sources is shown in Table 5. An advantage of the algorithm is that it reflects the degree of uncertainty related to the use of medication, diagnoses reported by GPs and self-report of disease or symptoms as measures for disease status. Moreover, combining data from various sources in an algorithm reduces the number of cases with missing data.

**Table 5:** Prevalence of diabetes mellitus at LASA wave C: Comparison between the revised diabetes mellitus algorithm and other data sources

Source	Definite	No	Possible	Contradictory	Missing
	n (%)	n (%)	n (%)	n (%)	n (%)
Self-reported^a^	199 (7.8)	2334 (91.7)	–	–	12 (0.5)
General practitioner	192 (7.5)	1794 (70.3)	37 (1.5)^c^	–	522 (20.5)
Medication^b^	89 (3.7)	1420 (55.8)	–	–	1036 (40.7)
Algorithm	180 (7.1)	2301 (90.4)	14 (0.6)	49 (1.9)	1 (0)

^a^Derived from face-to-face interview with respondent or telephone interview with respondent or proxy

^b^Based on ATC code A10 (drugs used in diabetes)

^c^Year of diagnosis was missing, therefore the diagnosis cannot be linked to a specific LASA wave

In 2018, the previously developed algorithms for CVD and diabetes mellitus were revised for several reasons. First, the algorithms could only be constructed for the B, C and D waves as some items on self-report of disease differed in content in later waves. Second, the use of certain medications is very disease specific, yet the diagnosis of the GP carried more weight in the former algorithms. This resulted in counter-intuitive diagnoses, for example a respondent who uses insulin was classified as ‘possibly’ having diabetes mellitus. For angina pectoris, cardiac arrhythmia and diabetes mellitus, the use of disease specific medication was given precedence over the other variables in the revised algorithms. Third, we have included data from the telephone interviews in the revised algorithms in order to reduce the number of cases categorized as missing. Chronic disease ascertainment algorithms are available for LASA waves B, C, D, E, 2B, F and G and will be developed for more recent waves as well.

Furthermore, algorithms for composite indices were constructed for the metabolic syndrome, allostatic load and frailty [25–29]. Metabolic syndrome and allostatic load algorithms are currently available for wave C only, but the metabolic syndrome algorithm can also be constructed for waves B and 2B (using antidiabetic medication as marker for fasting glucose) and for wave G. Algorithms for two widely used frailty constructs, the frailty phenotype (Fried criteria) and the frailty index (deficit accumulation approach) have been established from wave C onwards, combining self-reported and performance data [30–33].

Lastly, an algorithm for Persistent Cognitive Decline (PCD) was developed to identify participants with probable dementia [34]. For wave C to H, this was determined on the basis of: (a) decline in global cognition, measured by the MMSE (> 2 SD decline since previous wave); (b) the Informant Questionnaire on Cognitive Decline in the Elderly (IQCODE; score > 27); (c) interviewer-reported reasons for loss to follow-up (if reason was ‘dementia’); (d) data from GP registries, indicating that a diagnosis of dementia was reported by the GP and/or specialist; and (e) psychogeriatric nursing home admittance and the presence of dementia as a cause of death. Based on this algorithm, 3-year incidence of PCD in LASA was estimated to be about 3% [34]. More details on the disease and composite algorithms have been published on the LASA website (www.lasa-vu.nl).

### Environmental data

The LASA study has been included in two cohort consortia that focus on the relationship between the environment and health outcomes: the Geoscience and Health Cohort Consortium (GECCO; www.gecco.nl) [35] and the MINDMAP project (www.mindmap-cities.eu) [36]. In GECCO, various large-scale and ongoing cohort studies in the Netherlands have been enriched with a variety of existing, objectively measured, environmental data that were collected from different sources [35]. The MINDMAP project aims to identify the opportunities and challenges posed by urban environmental characteristics for the promotion and management of mental well-being and cognitive function of older adults [36]. This consortium brings together cohort studies across cities in Europe, Canada and the United States of America, and links these cohorts with databases of area-level environmental exposures and social and urban policy indicators. The environmental data collected for the GECCO consortium and MINDMAP project can be linked to individual LASA respondent data using their 4-digit postal codes or, where possible, 6-digit postal codes as the identifier.

The main environmental data that are currently available in LASA are shown in Table 6 and include: urbanization grade (i.e., number of residents/km^2^), population demographics (e.g., age- and sex-distribution, marital status and proportions of immigrants and ethnic minority groups), household characteristics (e.g., average household size), educational level (e.g., proportions of individuals who attained high, intermediate or low education), income (e.g., average income and proportion of income recipients), socioeconomic status (e.g., socioeconomic status score), social security (e.g., proportion of social security beneficiaries), air pollution (e.g., concentrations of air pollutants), noise (e.g., road-traffic, rail-traffic and air-traffic noise), crime rates (e.g., number of criminal offenses per 1000 residents), availability of facilities (e.g., in terms of density of/proximity to specific health care facilities and socio-cultural facilities), physical environmental characteristics (e.g., green space and water) and daily average weather parameters (e.g., daily average temperature and humidity). Several of these variables have been used in recent LASA studies [35, 37–41].

**Table 6:** Availability of environmental data in LASA

Environmental data	Spatial scale	Currently available for the years	Original data source
Urbanization grade	Neighborhood	1995, 1997, 1999, 2001, from 2003 to 2014	Statistics Netherlands
Population demographics	PC4	From 1998 to 2014	Statistics Netherlands
	Neighborhood	1995, 1997, 1999, 2001, 2003–2014	
Household characteristics	PC4	From 1998 to 2014	Statistics Netherlands
	Neighborhood	1995, 1997, 1999, 2001, 2003–2014	
Educational level	PC4	2014	Statistics Netherlands
Income	Neighborhood	1995, 1997, 1999, 2001, 2003, 2005, from 2009 to 2014	Statistics Netherlands
Socio-economic status score	PC4	1998, 2002, 2006, 2010, 2014	The Netherlands Institute for Social Research
Social security	Neighborhood	From 2003 to 2006, from 2008 to 2014	Statistics Netherlands
Air pollution	Addresses	2009	Institute for Risk Assessment Sciences
	PC6	2009	
	PC4	2009	
Noise	Addresses	2000, 2004, 2005, 2007, 2008, 2010, 2011	Netherlands Environmental Assessment Agency
	PC6	2000, 2004, 2005, 2007, 2008, 2010, 2011	
	PC4	2000, 2004, 2005, 2007, 2008, 2010, 2011	
Crime	Neighborhood	From 2010 to 2015	Statistics Netherlands
Availability of facilities	Neighborhood	From 2008 to 2014	Statistics Netherlands
Physical environment (green space, water)	Neighborhood	2006	Statistics Netherlands
Daily average weather parameters	Nationwide	From 2010 to 2012	The Royal Netherlands Meteorological Institute

This is an overview of the main environmental data currently available in LASA. More data may become available in the future. For a complete overview, see www.gecco.nl

*PC4* 4-digit postal code area, *PC6* 6-digit postal code area

## Migrant cohort

In 2013–2014, a sample of older adults born in Turkey and Morocco was included in LASA (wave MB). These migrants comprise the third and second largest groups of older non-Western migrants living in the Netherlands [42]. In the 1960s and 1970s, predominantly male Turkish and Moroccan migrants arrived in the Netherlands to perform (mostly) physical manual labor [43]. Later waves of migration from these countries took place in the 1980s when many wives and children from Turkey and Morocco rejoined their husbands living in the Netherlands. It was expected that these groups would face a number of additional challenges in older age, relative to their Dutch age-peers. Many face language barriers [44], unemployment [45], poverty [46], discrimination and prejudice [47]. On average, they are expected to experience more rapid health decline than their native peers [48, 49], have higher levels of loneliness and depression [50], and are in greater need of care [46]. By including a sample of Turkish and Moroccan migrants living in the Netherlands we aimed to investigate functioning in the domains of social, physical, emotional and cognitive functioning in these groups and to compare their functioning to that of Dutch age-peers. In addition, we aimed to study how characteristics of migration contributed to functioning in these domains.

### Migrant cohort sample and measurements

Data were collected among 478 older adults from Turkish (n = 269) and Moroccan (n = 209) origin with birth years between 1948 and 1957. The cooperation rate was 45%. Because Turkish and Moroccan migrants in the Netherlands predominantly live in urban areas, data collection took place in 15 Dutch cities with population sizes between 85,000 and 805,000 inhabitants. Specifically, the cities were Amsterdam, Zwolle, Oss, Alkmaar, Almere, Amersfoort, Breda, Eindhoven, Enschede, Haarlem, Helmond, Hilversum, Nijmegen, Tilburg and Zaanstad. Trained interviewers of the same ethnic background conducted face-to-face interviews in Dutch, Turkish, Moroccan Arabic (Darija) or Berber language (Tarifit). If available, translated questionnaires were obtained from prior studies, such as the De Jong Gierveld Loneliness Scale [51] and the Center for Epidemiologic Studies Depression scale (CES-D) [52]. If questionnaires were not available in Moroccan Arabic, Berber or Turkish, questions were translated by two professional translators according to the back-and-forth method. All questionnaires were evaluated and tested in pilot-interviews.

Data were collected in a main interview and in a subsequent medical interview (Table 1). Of the respondents who participated in the main interview (n = 478), a large part was also interviewed in a medical interview (n = 344, 72%). The main measures are listed in Table 7. No follow-up data have been collected among respondents included in the LASA migrant cohort. Data on mortality may become available in the near future, as vital status can be retrieved from municipality registers.

**Table 7:** Main measures in migrant cohort (wave MB, 2013–2014)

Measure	Details	Main interview	Medical interview
*Physical functioning*			
Body composition	Anthropometry		X
Lifestyle factors	Self-report		X
Chronic diseases	Self-report	X	X
Blood pressure	Blood pressure monitor		X
Functional limitations	Self-report	X	
Physical performance	Performance test	X	
Pain	Self-report		X
Falls/fractures	Self-report		X
Medication	ATC codes		X
Self-rated health	Self-report	X	
*Cognitive functioning*			
General cognitive functioning	MMSE (illiterate and literate)	X	
Executive functioning	Verbal fluency		X
Memory; memory complaints	Self-report	X	
*Emotional functioning*			
Depressive symptoms	CES-D		X
Personality trait	Mastery		X
*Social functioning*			
Contact network	Contact frequency	X	
Loneliness	De Jong Gierveld loneliness scale	X	
Social participation	Self-report	X	
*Other*			
Demographic and socio-economic factors	Self-report	X	
Religion, Religiosity	Self-report, RCOPE	X	
Use of care	Self-report	X	X
*Migrant specific*			
Family members’ residence	Self-report	X	
Visits Turkey/Morocco	Self-report	X	
Participation in organizations	Self-report	X	
Acculturation	TECI	X	
Length of residence in the Netherlands	Self-report	X	
Ethnic identity	Self-report	X	
Care use in Turkey/Morocco	Self-report	X	
Considering return migration	Self-report	X	

*ATC* Anatomical Therapeutic Chemical classification system, *MMSE* Mini Mental State Examination, *CES-D* Center for Epidemiologic Studies Depression scale, *RCOPE* Religious coping, *TECI* Taal en Cultuur Index [Language and Culture Index]

In the past few years, various studies using data from the LASA migrant cohort have been published. Two studies were conducted on transnational belonging [53, 54]. One study found that those feeling marginalized were lonelier and that transnational belonging was not protective of loneliness [53]. The other study investigated determinants of transnational behavior and transnational belonging. Family-in-laws’ location and gender explained transnational belonging, subjective income explained transnational behavior, and cultural distance and self-rated health explained both [54]. A study on wellbeing investigated whether private and public religious activities reduced the negative effects of a lack of physical, social and socio-economic resources on wellbeing. Private religious activities were positively associated with wellbeing but negatively associated with wellbeing in the context of lacking resources [55].

Three studies were performed in which the LASA migrant cohort was compared with native Dutch age-peers at wave 3B (2012–2013). First, a study on resilience in the disabling effect of physical impairments indicated that sense of mastery buffered against disability in those with physical impairment in the Turkish group. Income acted as a buffer against disability in those with physical impairment in the Dutch sample, but not in the migrant groups [56]. Second, measurement (in)variance of the CES-D was studied among older people of Dutch, Turkish and Moroccan origin, and the levels of depressive symptoms were compared. If the four sub-scales (i.e. depressed affect, positive affect, somatic symptoms and interpersonal problems) were used, scores were measurement invariant, which means that they measure the same construct across ethnic groups. However, migrants reported more depressive symptoms than native Dutch older adults on all sub-scales [57]. Finally, explanations for higher rates of loneliness among Turkish and Moroccan older adults compared to native Dutch older adults were examined. Less social participation, lower satisfaction with their income, poorer self-rated health and a higher number of depressive symptoms partially explained the higher rates of loneliness among migrants [58].

## Qualitative data collections

The rich resource of quantitative data in the LASA database provides the possibility to purposively select and approach subsamples of older adults for in-depth, qualitative research. Capitalizing on this strength several ancillary qualitative data collections have taken place to answer specific research questions. For example, in-depth interviews about the meaning and experience of control in health care have been conducted [59]. In addition to providing new insights into the factors that may enhance older adults’ sense of control, the conceptual model emerging from this qualitative study was subsequently used to develop a questionnaire measuring perceived control in health care [60]. Another qualitative study investigated resilience in older adults who aged successfully despite a low lifetime socioeconomic position (SEP) [61]. Previously calculated 16-year longitudinal trajectories of social, mental and physical functioning and three available indicators of SEP were used to identify the target group that was most likely to possess the experiences relevant to the research question. Furthermore, experiences of older Turkish and Moroccan migrants have been examined [62]. The life course experiences captured in the qualitative interviews provided insights into the aging experiences of migrants in the Netherlands, and the resources they use to cope with migration and aging related challenges. Finally, a photovoice study has been conducted among a purposively selected sample of LASA respondents living in the city of Amsterdam, who were asked to photograph aspects of their living environment that were important for their wellbeing. Photographs were then discussed in in-depth interviews to investigate the importance of the living environment for their wellbeing. This method enabled the researchers to capture a richer picture of the lived experiences and perceptions of older people. Besides, having participants decide for themselves what aspects they photographed gave them control over the contents and the direction of the interviews, which may empower participants [63].

These examples indicate that embedding qualitative ancillary studies in cohort studies such as LASA has several advantages. First, it enables researchers to answer questions on lay perceptions of aging and offers a ‘thick description’ of a topic of interest. Second, the results from qualitative studies can illustrate, complement and help to understand results from quantitative studies. For example, it may aid in developing new research ideas to be tested with quantitative methods, or in the development and implementation of new quantitative research instruments in the total LASA cohort.

One potential drawback of the ancillary qualitative studies is that they increase the burden on the participants, and this might negatively affect their subsequent participation in the ongoing study. However, our impression is that respondents welcome the variation in the mode of data collection. Additionally, the qualitative studies usually take place in a very small portion of the total sample (n < 30), and are thus unlikely to substantially affect overall response rates.

## Data Availability

LASA data are available for research. The LASA Steering Group has adopted a policy of sharing of data with interested researchers for specific research questions on aging-related issues. To obtain data, researchers need to submit an analysis proposal that is evaluated by the LASA Steering Group. Data are available for investigation under the condition that results of analyses will be made available to the research community through scientific reports or research papers, regardless of the results of the study. More information on data requests can be found on the LASA website: www.lasa-vu.nl. Forms to request assessment of biomarkers are also available here. We are open for collaborations in GWAS meta-analysis and gene-environment interaction studies. A specific analysis proposal format for GWAS meta-analysis is available on the website as well.
